# Characterization, Large-Scale HSCCC Separation and Neuroprotective Effects of Polyphenols from *Moringa oleifera* Leaves

**DOI:** 10.3390/molecules27030678

**Published:** 2022-01-20

**Authors:** Qian Gao, Zongmin Wei, Yun Liu, Fang Wang, Shuting Zhang, Carmo Serrano, Lingxi Li, Baoshan Sun

**Affiliations:** 1 School of Functional Food and Wine, Shenyang Pharmaceutical University, Shenyang 110016, China; 15148993690@163.com (Q.G.); abcd13363316067@163.com (Y.L.); wangf@syphu.edu.cn (F.W.); zstzwz-3@163.com (S.Z.); 2 School of Traditional Chinese Materia Medical, Shenyang Pharmaceutical University, Shenyang 110016, China; weizm_12345678@163.com; 3 Jiangsu Hansoh Pharmaceutical Group Co., Ltd., Lianyungang 222069, China; 4 Unidade de Tecnologia e Inovação, Instituto National de Investigação Agrária e Veterinária, 2780-157 Oeiras, Portugal; carmo.serrano@iniav.pt; 5 Pólo Dois Portos, Instituto National de Investigação Agrária e Veterinária, I.P., Quinta da Almoinha, 2565-191 Dois Portos, Portugal

**Keywords:** *Moringa oleifera* leaf, high-speed countercurrent chromatography, polyphenols, quercetin, kaempferol, neuroprotective effects

## Abstract

*Moringa oleifera* leaves have been widely used for the treatment of inflammation, diabetes, high blood pressure, and other diseases, due to being rich in polyphenols. The main objective of this work was to largely separate the main polyphenols from *Moringa oleifera* leaves using the technique of high-speed counter-current chromatography (HSCCC). The phenolic composition in *Moringa oleifera* leaves was first analyzed qualitatively and quantitatively by UPLC-Q-Exactive Orbitrap/MS and UPLC-QqQ/MS, respectively, indicating that quercetin and kaempferol derivatives, phenolic acid and apigenin are the main polyphenols in *Moringa oleifera* leaves, with quercetin and kaempferol derivatives predominating. Furthermore, the conditions of HSCCC for large-scale separation of polyphenols from *Moringa oleifera* leaves were optimized, which included the selection of the solvent system, flow rate and the sample load. Only by one-step HSCCC separation (within 120 min) under the optimized conditions, six quercetin and kaempferol derivatives, a phenolic acid and an apigenin could be individually isolated at a large scale (yield from 10% to 98%), each of which possessed high purity. Finally, the isolated polyphenols and phenolic extract from *Moringa oleifera* leaves (MLPE) were verified to have strong neuroprotective activities against H_2_O_2_-induced oxidative stress in PC-12 cells, suggesting that these compounds would contribute to the main beneficial effects of *Moringa oleifera* leaves.

## 1. Introduction

*Moringa oleifera* Lam. belongs to the Moringaceae family of perennial tropical deciduous trees, which contains more than 12 species [1] and is a single-genus family of shrubs [2], named by French naturalist Jean-Baptiste Lamarck. All Moringa species originated in Asia were introduced to other warm countries from there. *Moringa oleifera* is called the “magical tree” by scientists [3], and has attracted more and more attention due to its abundant nutrients and biological metabolites [4]. In Indian traditional medicine, *Moringa oleifera* was used to prevent and treat more than 300 diseases [5,6,7,8,9,10,11]. Each part of *Moringa oleifera* has shown medicinal value, and the leaves of *Moringa oleifera* are the most extensively studied. *Moringa oleifera* leaf holds some certain therapeutic potential for many diseases, for instance, chronic hyperglycemia, hyperlipidemia [12], immune regulation [13], hypertension [14], cancer [15], liver disease, inflammation, etc. [16,17,18,19,20,21,22,23]. The natural active material of *Moringa oleifera* leaf contain polyphenols, polysaccharides, and alkaloids, etc., among which polyphenols occupy an important position and are important secondary metabolite products. A previous study discovered that polyphenols from *Moringa oleifera* leaf extract exhibited a neuroprotective effect and can be claimed as a therapeutic target for neurogenic disease [24].

*Moringa oleifera* leaf polyphenols mainly contain flavonoids and phenolic acids. The research results by Coppin et al. [25] showed that *Moringa oleifera* leaves contain at least 12 flavonoids, consisting of quercetin, kaempferol glucosides and glucoside malonates as the main components. Kashiwada et al. [26] isolated two new caffeoylquinic acid glucosides together with three known caffeoyl quinic acids and five known flavonoid glucosides from *Moringa oleifera* leaves. In order to analyze the composition of polyphenols in *Moringa oleifera* leaves, some researchers used traditional separation methods to isolate and purify polyphenols [27,28,29,30]. Kashiwada et al. adopted column chromatography packed with MCI gel CHP20P, Sephadex LH-20 and YMC ODS-A for the separation of polyphenols. Cao et al. [31] employed column chromatography with five different types of macroporous resin to purify *Moringa oleifera* seed polyphenols. Although these two studies have achieved the separation of the target compounds and the effect of purification to a certain extent, this separation method showed some disadvantages such as tedious operation, long period, the irreversible adsorption of samples and low yields. Abhishek Niranjan et al. [32] used high-performance thin-layer chromatography to separate six phenols in the different parts of *Moringa oleifera*. Although the separation of the main phenolic substances was realized, this method also had some disadvantages, such as complex operation, large artificial impact and poor reproducibility. Other conventional separation and purification methods, e.g., organic reagent extraction, membrane separation and precipitation separation, have also been applied for the separation of *Moringa oleifera* polyphenols [33,34]. However, these methods are accompanied with some disadvantages such as being uncontrollable, low yield, safety risks in the introduction of foreign substances, complex purification process, being environmentally unfriendly and so on [35]. The drawbacks of these traditional separation and purification methods limit the analysis and development of *Moringa oleifera* leaf polyphenols [36]; therefore, it is undoubtedly important to develop a more effective method for the large-scale preparation of these bioactive compounds in *Moringa oleifera* leaves.

During the last few years, with the intention of reducing the amount of solvent required, as well as sample preparation costs and operating time, a number of updated extraction and separation techniques have been proposed. Among them, high-speed counter-current chromatography (HSCCC) is a typical approach. It is a liquid–liquid partition chromatography technology that does not use a solid support matrix, meaning that it is able to eliminate the irreversible adsorption loss of the sample caused by the solid support matrix in the traditional chromatography column [37,38]. Additionally, it has the merits of simple pretreatment operation, high reproducibility, and high recovery rate [39]. The applications of this approach have been widely used in the separation and purification of a variety of active ingredients from natural products [40,41,42,43,44,45,46]. In our previous studies, HSCCC was successfully applied to separate and isolate polyphenols at a large scale from different plants [47,48,49]. The results indicate that this emerging separation technology is convenient and appears to be an efficient strategy for polyphenol separation.

The purpose of this study was to characterize the phenolic composition of *Moringa oleifera* leaves using UPLC-Q-Exactive Orbitrap/MS and UPLC-QqQ/MS and to develop a method using HSCCC to largely separate the main polyphenols from *Moringa oleifera* leaves. Moreover, the neuroprotective effects of phenolic extract and two representative polyphenols, quercetin and kaempferol, of the *Moringa oleifera* leaves were evaluated in a cellular experiment.

## 2. Results and Discussion

### 2.1. Optimization of UPLC Conditions

An updated approach for determining phenolic composition in MLPE was carried out. Different mobile phases (including methanol:water, acetonitrile:water, acetonitrile–formic acid:water and methanol–formic acid:water), column temperatures (10, 20, 30 and 40 °C), and flow rates (0.1, 0.2 0.3 and 0.4 mL/min) were determined and compared. With the intention of performing this optimization, the mobile-phase gradient and its flow rate were selected based on our prior experiments. The optimal gradient was generated by a number of trial-and-error tests. When the compounds achieved the optimal separation effect, the focus then shifted to decreasing analysis time.

As a result, according to the final outcomes, the most minor pressure change, the shortest analysis time, and the greatest resolution could be obtained when the flow rate of the mobile phase was 0.3 mL/min, and the column temperature was 30 °C. In addition, the gradient elution program adopted two elution solvents, A (water: formic acid; 99.9:0.1, *v*/*v*) and B (acetonitrile): 0 min (A 95%:B 5%), 10 min (A 75%:B 25%), and 18 min (A 0%:B 100%).

### 2.2. Optimization of MS Conditions

To establish sensitive and accurate qualitative and quantitative methods, the MS (Q-Exactive Orbitrap/MS) and the MS/MS (QqQ/MS) fragmentation patterns were investigated. Firstly, the Q-Exactive Orbitrap/MS spectra in negative and positive ion modes were studied and compared. The results indicate that the negative ion mode was more sensitive and selective for phenolic composition in *Moringa oleifera* leaves. Therefore, the negative ion mode was used in Q-Exactive Orbitrap/MS and QqQ/MS analysis. To obtain the most intense transition for quantitation, the CE and source cone voltages (CV) of standards and MLPE were optimized. The optimized values for the eight target compounds are shown in Table 1.

### 2.3. Identification of Individual Phenolic Compounds in MLPE

Under the optimized conditions, phenolic components in MLPE were analyzed by UPLC-Q-Exactive MS/MS in negative mode. In total, 22 different classes of phenolic compounds were identified, which included 10 flavonols (4 quercetin derivatives, 4 kaempferol derivatives, 2 apigenin derivatives), 8 phenolic acids and 4 others, of which quercetin and kaempferol derivatives were the two main compounds in *Moringa oleifera* leaves. Table 2 lists these phenolic compounds along with their retention time, molecular formula, theoretical mass, observed and calculated masses and MS/MS fragments.

#### 2.3.1. Flavonols

The flavonols in MLPE were identified by Q-Exactive Orbitrap/MS under negative ion mode. As shown in Table 2, flavonols in *Moringa oleifera* leaves were mainly composed of four quercetin derivatives, four kaempferol derivatives and two apigenin derivatives. The cleavage rules of flavonol compounds are as follows: aglycone ions usually undergo cleavage, loss and rearrangement centered on the C-ring in the positive and negative ion mode [46]. The carbon–carbon bonds at the positions of the C-ring 1/3, 0/2 and 0/4 can all be broken, and the fractures at the positions of 1/3 and 0/4 are RetmDid-Alder (RDA) cleavage [50]. In addition, aglycone ions usually produce a series of cleavages of neutral fragments such as CO, CO_2_ and H_2_O in positive and negative ion mode [51]. The fragmentation of oxyglycosides by mass spectrometry mainly includes glycosidic bonds and the cross-ring cleavage of the glycosyl ring to form aglycone ions. The mass spectrometric cleavage of carbon glycosides is quite different from that of oxygen glycosides, which is mainly caused by the removal and cleavage of the cross ring of the sugar ring, and the corresponding aglycone ions are almost not formed [52].

Compound 14 gave [M–H]^−^ ions at *m*/*z* 609.1464 and the relative molecular weight could be calculated to be 610. The characteristic ion fragments of the second-order mass spectrometry are *m*/*z* 300.0276 and 343.0455, respectively. It was consistent with rutin, as described by Makita et al. [53], so compound 14 was identified as rutin. Compound 15 showed [M–H]^−^ ions at *m*/*z* 463.0887 and the relative molecular weight could be calculated to be 464. The characteristic ion fragments of the second-order mass spectrometry are *m*/*z* 300.0276 and 343.0464, respectively. It was consistent with quercetin-3-O-glucoside, as described by Yan et al. [54], so compound 15 was identified as quercetin-3-O-glucoside (isoquercitrin). Compound 16 gave [M–H]^−^ ion at *m*/*z* 505.0993 and the MS/MS fragment ions showed the characteristic fragmentation ion at *m*/*z* 300.0276 and *m*/*z* 343.0455 (loss of 162 Da), which was identified as quercetin-acetyl-glycoside [55]. Similarly, compound 17 was identified as quercetin-malonyl-glucoside [56].

Kaempferol derivatives were also identified in MLPE. Compound 18 presented characteristic fragments at *m*/*z* 593.1516 ([M–H]^−^), *m*/*z* 327.0510 and *m*/*z* 285.0405 and was tentatively identified as kaempferol-3-O-rutinoside [53]. Compound 20 presented characteristic fragments at *m*/*z* 447.0936 ([M–H]^−^), *m*/*z* 284.0327 and *m*/*z* 327.0513 and was tentatively identified as astragalin [57]. Compound 21 presented characteristic fragments at *m*/*z* 489.1042 ([M–H]^−^), *m*/*z* 284.0328 and *m*/*z* 327.0495 and was tentatively identified as kaempferol-acetyl-glycoside [54]. Compound 22 presented characteristic fragments at *m*/*z* 533.1724 ([M–H]^−^), *m*/*z* 255.0295 and *m*/*z* 285.0404 and was tentatively identified as kaempferol-malonyl-glycoside [56].

Similarly, compound 12 presented characteristic fragments at *m*/*z* 593.1516 ([M–H]^−^), *m*/*z* 353.0668, *m*/*z* 383.0775and *m*/*z* 473.1091 and was tentatively identified as vicenin-2 [58], Additionally, compound 13 presented characteristic fragments at *m*/*z* 431.0984 ([M–H]^−^), *m*/*z* 283.0614, *m*/*z* 311.0563 and *m*/*z* 341.0666 and was tentatively identified as vicenin [56].

#### 2.3.2. Phenolic Acids and Other Polyphenols

Phenolic acids in MLPE under negative ion mode were also detected by Q-Orbitrap/MS analysis. As shown in Table 2, a total of eight phenolic acids and four others were identified.

Compounds 1, 2, 3, 6, 8, 9, 10 and 11 were phenolic acids and their derivatives. Compound 1 presented characteristic fragments at 178.9769 ([M–H]^−^), *m*/*z* 161.0444 and *m*/*z* 89.0228. It was identified as caffeic acid. The MS data were in accordance with the reference standard [59]. Compound 2 presented characteristic fragments at *m*/*z* 190.9276 ([M–H]−), *m*/*z* 85.0278 and *m*/*z* 127.0387 and was tentatively identified as quinic acid. Compound 3 presented characteristic fragments at *m*/*z* 133.0129 ([M–H]^−^), *m*/*z* 71.0122 and *m*/*z* 115.0022 and was tentatively identified as malic acid.

Compound 6 and 8 presented characteristic fragments at *m*/*z* 353.0880 ([M–H]^−^), *m*/*z* 135.0437, *m*/*z* 179.0340 and *m*/*z* 191.0552, and were tentatively identified as caffeoylquinic acid. The MS data were in accordance with the reference standard [60]. Therefore, we inferred that compound 6 and 8 were isomers of caffeoylquinic acid, and should be mono-caffeoylquinic acid according to the molecular weight, so they were tentatively identified as 4-caffeoylquinic acid and 3-caffeoylquinic acid. Compounds 9 and 10 presented characteristic fragments at *m*/*z* 377.0931 ([M–H]^−^), *m*/*z* 119.0488, *m*/*z* 163.0389 and *m*/*z* 191.0553, and were tentatively identified as 4-coumaroylquinic acid and 3-coumaroylquinic acid [61]. Compound 11 presented characteristic fragments at *m*/*z* 367.1036 ([M–H]^−^), *m*/*z* 134.0359 and *m*/*z* 193.0498 and was tentatively identified as feruloylquinic acid.

Compound 4 presented characteristic fragments at *m*/*z* 570.0964 ([M–H]^−^), *m*/*z* 96.9585 and *m*/*z* 259.0131 and was tentatively identified as glucomoringin [62]. Compounds 5 and 7 presented characteristic fragments at *m*/*z* 315.0724 ([M–H]^−^), *m*/*z* 108.0201, *m*/*z* 89.0228, *m*/*z* 123.0438 and *m*/*z* 153.0545, and was tentatively identified as vanillin glucoside isomer, and compound 19 presented characteristic fragments at *m*/*z* 521.2034 ([M–H]^−^), *m*/*z* 101.0228 and *m*/*z* 341.1398 and was tentatively identified as isolariciresinol glucoside [63].

### 2.4. Method Validation

The calibration curves of each standard were constructed with seven concentration levels, and each concentration was set in triplicate. All curves revealed a good linear relationship (r^2^ > 0.999) in the experimental concentration range (Table 3). The lowest values of LOD and LOQ both corresponded to astragalin (0.01046 and 0.03486 μg/mL, respectively) and the highest ones to 4-caffeoylquinic acid (0.05388 and 0.1796 μg/mL, respectively).

Under the conditions established, the RSDs of the measured indicators of standards were all less than 5.0% (Table 4), and the recoveries ranged from 95.2% to 104.9% (Table 5) with RSDs less than 4.1%. The above data (Table 3, Table 4 and Table 5) were deemed to be satisfactory for the quantification of all standards and MLPE.

The method established had a good linearity, accuracy, precision, sensitivity and stability, and can be used for the quantification of polyphenols of *Moringa oleifera* leaves.

### 2.5. Quantification of Phenolic Compounds in MLPE

To obtain the detailed phenolic composition in MLPE and to better calculate the yield of polyphenols obtained by HSCCC, a precise quantitative analysis of the main polyphenols from *Moringa oleifera* leaves identified by QqQ/MS was performed. A total of eight compounds in the MLPE were quantitatively analyzed.

The validated method was used to quantify eight polyphenols in MLPE. The internal standard method was used to quantify eight compounds in MLPE based on calibration curves. Quercetin-acetyl-glycoside was quantified with the calibration curve of isoquercitrin. Kaempferol-malonyl-glycoside was quantified with the calibration curve of kaempferol-3-O-rutinoside. The quantitative results of eight compounds are presented in Table 6.

As we can see from Table 6, the content of quercetin-acetyl-glycoside in the quercetin derivatives (2.456 ± 0.004 mg/g) of the MLPE was higher than others. There is currently no commercial standard for the substance, and as far as we know, quercetin-acetyl-glycoside has only been found in *Moringa oleifera*. The contents of isoquercitrin and rutin were 2.293 ± 0.005 mg/g and 2.267 ± 0.006 mg/g, respectively. Unfortunately, the content of them in this study was lower than that studied by Edith N. Fombang et al. [64], which may be due to the differences in geographic regions and the treatment methods of the raw materials of *Moringa oleifera* [65]. However, a common observation between the two studies was that the content of rutin in *Moringa oleifera* leaves was higher than isoquercitrin. The content of kaempferol-malonyl-glycoside (0.1254 ± 0.003 mg/g) in the kaempferol derivatives was higher than others. It is gratifying that there is no commercial standard for this substance at present. The contents of kaempferol-3-O-rutinoside and astragalin of the other two kaempferol derivatives were 1.206 ± 0.003 mg/g and 0.4007 ± 0.002 mg/g, respectively. The results of Li et al. [66] show that *Ginkgo biloba* L. male flowers were rich in kaempferol-3-O-rutinoside, but the content was lower than that in *Moringa oleifera* leaves, although astragalin was the least abundant substance in kaempferol derivatives. Moreover, 4-caffeoylquinic acid and a small amount of vitexin were also isolated by HSCCC, and the contents were 1.985 ± 0.008 mg/g and 0.3415 ± 0.005 mg/g, respectively. In this study, the content of vitexin in all substances was the lowest (0.3415 ± 0.005 mg/g). Studies by Kalinová et al. [67] showed that vitexin was found in many plants, among which buckwheat (*Buckwheat*) had the highest content of vitexin (about 3.548 mg/g), which was higher than that in the raw materials of this experiment. However, compared with most other plants (such as *flax*, *linseed*, *fenugreek*, etc.), the content of vitexin in the leaves of *Moringa oleifera* was still higher than others, indicating that the leaves of *Moringa oleifera* can be used as a high-quality source of vitexin.

UPLC-QqQ/MS is a powerful quantitative system for the quantification of polyphenols in MLPE. The applicability of the method established in this study was verified in terms of linearity, repeatability, precision and stability. Additionally, these results also suggest that *Moringa oleifera* leaves are a high-quality source of polyphenols.

### 2.6. Optimization of HSCCC Conditions

Quercetin and kaempferol derivatives are the two main polyphenols of *Moringa oleifera* leaves, both of which have a variety of biological activities. The main objective of this part was to largely separate the main polyphenols (quercetin and kaempferol derivatives) from *Moringa oleifera* leaves using HSCCC.

Based on the structural characteristics, solubility and polarity of quercetin derivatives and kaempferol derivatives, and the average polarity of the solvent system, a series of biphasic solvent systems using HEMWat were investigated, as shown in Table 7.

As discussed above, selecting a suitable solvent system is one of the most significant steps during the process of successful HSCCC separation, and the partition coefficient (*K*) value is the most vital indicator for determining the resolution in HSCCC. When the *K* value is less than 0.5, the material to be separated is washed out too fast, which easily leads to low resolution; when the *K* value is higher than 2, the peak time of the material is too long, which easily leads to the broadening of the peak shape and the decrease in separation efficiency. Separation factor (α) is an important indicator for judging the degree of separation of two substances. A larger α shows greater separation between peaks [68]. Therefore, in order to better separate isoquercitrin and astragalin, *K* and α are used to determine the optimal solvent composition. Additionally, the range of *K* values applicable to HSCCC should be between 0.5 and 2.0; in the meantime, the α between two elements is required to be higher than 1.2 (α = *K*_2_/*K*_1_, *K*_2_ > *K*_1_).

Table 7 displays the *K* values and the corresponding α of isoquercitrin and astragalin. The *K* values of the solvent systems of H_2_O-hexane-ethyl acetate (50:1:50, *v*/*v*), HEMWat (3:5:3:5, *v*/*v*) and HEMWat (1:1:1:1, *v*/*v*) are more than 20, indicating that these solvent systems had significant retention of the stationary phase, and the sample was difficult to wash off. The *K* values of the other three solvent systems were all in the suitable range, and close to the ideal value of 1 [45], which is the prerequisite for the separation of isoquercitrin and astragalin. The α values of the solvent systems of HEMWat (1:3:1:3, *v*/*v*) and HEMWat (1:5:1:5, *v*/*v*) were greater than 1.2, which indicates that the separation resolutions of these two solvent systems were feasible. Finally, after the trial and comparison of the two solvent systems, HEMWat (1:5:1:5, *v*/*v*) was considered to be the most appropriate solvent system for this study due to its better peak shape.

Other factors were analyzed as well, including the flow rate of the mobile phase and the sample load. The flow rate of the mobile phase has a direct contribution to the amount of stationary phase fixed in the column and the separation time, and as a consequence, affects the peak resolution [68]. In this study, the flow rates of 2.0 mL/min and 3.0 mL/min were analyzed at the revolution speed of 950 rpm, individually. The analysis results indicate that when the flow rate of 2.0 mL/min was applied, the detection time was extended and the peak shape was unsightly, and the flow rate of 3.0 mL/min did not perform well in terms of separation resolution and stationary phase retention. As a result, this study chose 2.5 mL/min to be the most appropriate flow rate of the mobile phase due to its shorter separation time (120 min), preferable peak resolution and higher retention of the stationary phase (S_f_ = 54.7%).

One of the most important goals for HSCCC separation was to obtain as many products as possible [69]. Therefore, the sample loading of 100 to 500 mg of the MLPE in the upper phase of 10 mL was analyzed as well. According to the analysis results, however, with the increase in the sample size, the elution band of the target compound began to widen [70]. Excessive sample loading may result in a massive loss of stationary phase, as well as a lower resolution in the chromatographic column, although little sample loading may lead to a reduction in product yield [71]. Therefore, this study selected 100 mg of MLPE as it contributed to lesser stationary phase loss and greater output. On the basis of these outcomes, the HSCCC separation was carried out by making use of the two-phase solvent system containing HEMWat (1:5:1:5, *v*/*v*) in head-to-tail elution modes. At the same time, the sample loading was set as 100 mg and the flow rate was set as 2.5 mL/min.

### 2.7. Preparative HSCCC Separation of MLPE Phenolic Compounds

In this work, a one-step HSCCC was utilized to separate the MLPE. When the HSCCC conditions were optimized, MLPE can be easily isolated into four fractions within 120 min of elution.

**Figure 1:**
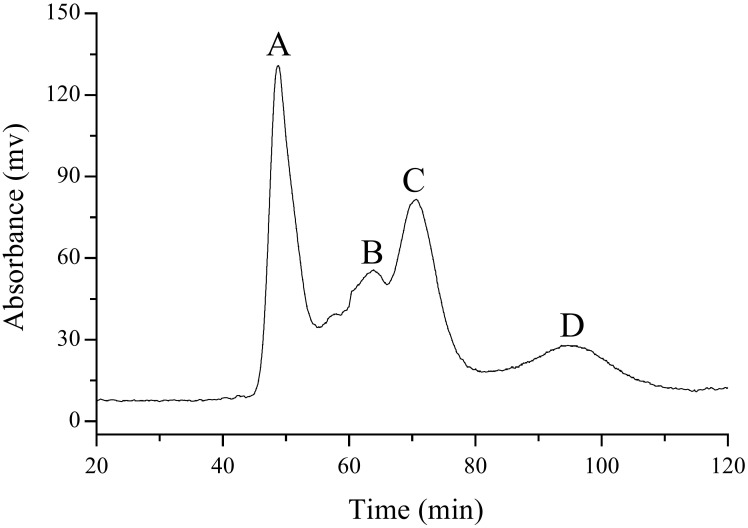
HSCCC chromatograms of preparative separation of polyphenols from MPLE under the optimized conditions, and four components were separated into **A**, **B**, **C** and **D**.

Figure 1 presents the HSCCC chromatogram, in which the peak shape of each component is relatively sightly, and a preferable resolution can be obtained. The peak curve of each component is smooth, and the repeatability of the peak shape is satisfactory, which is better than most of the previous traditional HSCCC peak patterns [72,73]. Particularly, fraction C and fraction D can achieve baseline separation. Additionally, the results indicate that sample loading can generate an average of 0.28 mg of fraction A, 0.088mg of fraction B, 0.185 mg of fraction C and 0.036 mg of fraction D. The peak curve of each component is smooth, and the repeatability of the peak shape is satisfactory, which is better than most of the previous traditional HSCCC peak patterns. Fractions A–D were analyzed by UPLC–MS and identified by MS identification.

Under the optimal conditions of HSCCC, the separation of MLPE was tested 5 times to calculate the repeatability. The relative standard deviation (RSD) values of repeatability of the fractions A–D were 5.6%, 4.9%, 3.4%, and 4.5%, respectively. The high repeatability of the apparatus and high-purity fractions C and D permitted follow-up active experiments.

### 2.8. UPLC–MS Analysis and MS Identification of Individual Polyphenols

The identification of the A-D fraction isolated by HSCCC was according to the fragmentation regularity from the electron impact mass spectra of flavonols [50,51,52,53], and the literature on related compounds.

#### 2.8.1. UPLC–MS Analysis of HSCCC Fractions

**Figure 2:**
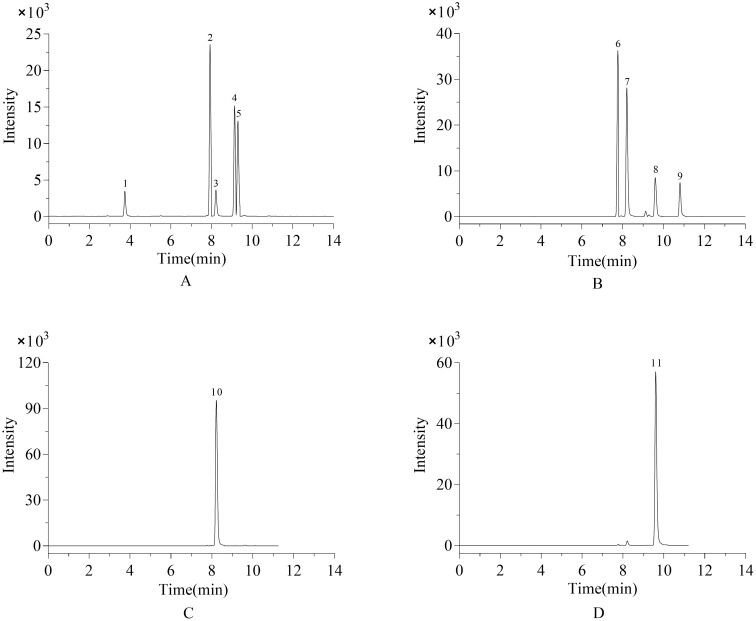
UPLC–MS analysis of Fractions (**A**–**D**) and MS identification of individual polyphenols. Fraction (**A**): peak 1, 4-caffeoylquinic acid; peak 2, rutin; peak 3, isoquercitrin; peak 4, quercetin-acetyl-glycoside; peak 5, kaempferol-3-o-rutinoside. Fraction (**B**): peak 6, vitexin; peak 7, isoquercitrin; peak 8, astragalin; peak 9, kaempferol-malonyl-glycoside. Fraction (**C**): peak 10, isoquercitrin. Fraction (**D**): peak 11, astragalin.

Figure 2A shows the chromatogram of UPLC–MS analysis of fraction A isolated from HSCCC. UPLC–MS analysis showed that, for fraction A, four compounds (compound 1, 2, 4 and compound 5) with higher polarity and a small amount of compound 3 were eluted firstly. Among them, the contents of compound 2, compound 4, and compound 5 were higher in fraction A, accounting for 37.94%, 25.51% and 23.69%, respectively. Figure 2B shows the chromatogram of UPLC–MS analysis of fraction B. UPLC–MS analysis showed that compounds 6–9 displayed similar elution behavior and were eluted together as the second fraction by HSCCC. That is, the polarity of fraction B was slightly lower than fraction A. Among them, compounds 6 and 7 accounted for a larger proportion in fraction B, reaching 37.91% and 37.48%, respectively.

Figure 2C,D displays the chromatograms achieved by the UPLC–MS analysis, which was conducted on fractions C and D, isolated from HSCCC. UPLC–MS analysis showed that compound 10 and compound 11 were the two main phenolic compounds of fractions C and D, respectively. The purity of each compound was checked by UPLC, and the results show that through one-step HSCCC separation, 0.185 mg of fraction C and 0.036 mg of fraction D with 89.4% and 81.4% purity could be generated from MLPE.

Both MLPE and target compounds have good solubility in medium polar solvents, so the sample was dissolved in the upper phase solution, and the lower phase with greater polarity was used as the mobile phase. Due to the diversity of groups, there was a certain interaction among the MLPE, upper phase and lower phase. Hence, with the elution of the mobile phase, a small number of target compounds immediately following some polar substances may be eluted, as shown in Figure 2 fraction B. However, owing to the stronger hydrophobicity of compound 10 and 11 than others, and the suitable solvent system, most compounds in the MLPE could be eluted in fractions C and D. In addition, a better degree of separation could be obtained.

#### 2.8.2. Structural Identification

By comparison with existing standards or our library data, compounds were identified according to their mass fragment characteristics and retention time. The results are listed in Table 8. Peak 1 presented characteristic fragments at *m*/*z* 353.0880 ([M–H]^−^), *m*/*z* 179.0340 and *m*/*z* 191.0552. It was identified as 4-caffeoylquinic acid due to the secondary fragments *m*/*z* 191.0552 and *m*/*z* 179.0340, which are fragment ions that have lost quinic acid and caffeic acid, respectively. The MS data were in accordance with the reference standard [60]. Peak 2 presentsed characteristic fragments at *m*/*z* 609.1464 ([M–H]^−^), *m*/*z* 300.0276 and *m*/*z* 343.0455 and was tentatively identified as rutin [53]. Peaks 3, 7 and 10 presented characteristic fragments at *m*/*z* 463.0887 ([M–H]^−^), *m*/*z* 300.0276 and *m*/*z* 343.0464 and were tentatively identified as isoquercitrin [54]. Peak 4 presented characteristic fragments at *m*/*z* 505.0993 ([M–H]^−^), *m*/*z* 300.0276, *m*/*z* 343.0455 and *m*/*z* 463.0896 and was tentatively identified as quercetin-acetyl-glycoside [55]. Peak 5 presented characteristic fragments at *m*/*z* 593.1516 ([M–H]^−^), *m*/*z* 285.0405 and *m*/*z* 327.0510 and was tentatively identified as kaempferol-3-O-rutinoside [53]. Peak 6 presented characteristic fragments at *m*/*z* 431.0984 ([M–H]^−^), *m*/*z* 283.0614, *m*/*z* 311.0563 and *m*/*z* 341.0666 and was tentatively identified as vitexin [56]. Peaks 8 and 11 presented characteristic fragments at *m*/*z* 447.0936 ([M–H]^−^), *m*/*z* 284.0327 and *m*/*z* 327.0513 and were tentatively identified as astragalin [57]. Peak 9 presented characteristic fragments at *m*/*z* 533.1724 ([M–H]^−^), *m*/*z* 255.0295 and *m*/*z* 285.0404 and was tentatively identified as kaempferol-malonyl-glycoside [56].

#### 2.8.3. Calculation of the Yield of Individual Polyphenols Obtained by HSCCC

The structural identification of HSCCC fractions results suggests that *Moringa oleifera* leaves are a high-quality source of quercetin and kaempferol derivatives, and they can be separated effectively in one step by HSCCC.

About 30% quercetin-acetyl-glycoside was isolated from *Moringa oleifera* leaves by using the optimized solvent system, which provided the experimental basis for further study on the activity of quercetin-acetyl-glycoside. Additionally, the total yields of isoquercitrin and rutin were 94% and 47%, respectively. Additionally, about 84% of kaempferol-malonyl-glycoside could be isolated from *Moringa oleifera* leaves in this study, which provided an effective preparation method for the commercialization of this substance. The total yield of astragalin obtained by high-speed countercurrent separation was over 95%, indicating that the optimized solvent system was very suitable for the preparation of astragalin. The yield of another kaempferol derivative (kaempferol-3-O-rutinosideobtained) was 55%. The yield of vitexin by the HSCCC method can be as high as 98%. In addition, 10% 4-caffeoylquinic acid was isolated.

Among the eight substances isolated, a large proportion of isoquercetin (yield of 94%) and astragalin (yield of over 95%) in the MLPE were eluted separately to fraction C and fraction D, with a purity of 89.4% and 81.4%, respectively. In spite of the fact that they are widely distributed in nature, their content in plants is very low (isoquercitrin is only 0.0067 mg/100 g–41.95 mg/100 g); therefore, it is difficult to obtain a large number of individual compounds with high purity in the food and pharmaceutical industries [74]. Additionally, no research has yet been reported on the preparation by chemical synthesis, which greatly limits the application of the two substances. Therefore, it is urgent to develop a more effective, quick and large-scale preparation method. The results of this study show that high-purity isoquercetin and astragalin can be prepared by HSCCC technology using *Moringa oleifera* leaves as raw materials, which provided a material basis for the follow-up study of their activity.

The main polyphenols in the MLPE were separated by HSCCC. A total of eight compounds were identified, which includes three quercetin derivatives and three kaempferol derivatives. In addition, a phenolic acid (4-caffeoylquinicacid) and an apigenin (vitexin) were isolated. Although most of these phenolic compounds could be obtained from other plants, most of them are barely commercially available or expensive. What is worthy of surprise was that among the eight substances isolated in this study, there were two non-commercial standard substances, kaempferol-malonyl-glycoside and quercetin-acetyl-glycoside. This work provides an effective method for the separation and preparation of the two substances. These results likewise demonstrate that *Moringa oleifera* leaves could be used as a source of quercetin and kaempferol derivatives.

Although it has been reported that several polyphenols have been isolated from other plants such as cotton petal by HSCCC, the sample pretreatment and preparation processes were tedious [75]. Especially time-consuming, this method took 2–3 times longer than that of this research study. Compared with the traditional separation methods, such as column chromatography, the yields of compounds 10 and 11 obtained by HSCCC were up to 4–5 times greater under the same separation time [76]. The bioguided assay was used to separate polyphenols from *Moringa oleifera* leaves [77], but the yields of isoquercitrin and astragalin were merely 1.9% and 0.17%, which were also far lower than the yields in this experiment. Similarly, compared with previous studies on the separation of two kinds of derivatives [78], this work had obvious advantages, such as fewer sample treatment steps and a simple separation process, that is, one-step separation, and four components can be obtained in one elution mode, including two individual compounds with high purity, and more target compounds can be effectively separated in the appropriate separation system. Other solvent systems, such as N-hexane-ethylacetate-n-butanol-water [79], have been applied to the separation of kaempferol and quercetin derivatives in *Sorbus tianschanica*. It had similar polarity and separation behavior to this research system. However, the number of compounds obtained by this solvent system was less than the system selected in this study, which means that HEMWat was more suitable for the separation and purification of the main polyphenols (kaempferol and quercetin derivatives) in *Moringa oleifera* leaves under the premise of simpler sample pretreatment.

In conclusion, this research proposed an updated HSCCC method for rapidly isolating the main polyphenols from MLPE on a large-scale, which is economical, time saving and highly efficient. It could be widely used for further bioactivity study.

### 2.9. Effect of Polyphenols in MLPE against H_2_O_2_-Induced Oxidative Stress in PC-12 Neuroblastoma Cells

The appropriate amount of reactive oxygen species (ROS) has an important role in maintaining normal cell signal transduction and cell metabolism [80]. Excessive ROS can lead to oxidative stress, which leads to a series of diseases, including many neurodegenerative diseases, such as AD and PD [81,82]. Therefore, it may be a potential strategy to protect the body against oxidative stress by using effective antioxidants.

Polyphenols have been recognized as natural antioxidants. *Moringa oleifera* leaves, which are rich in polyphenols, have been verified as safe with no reported adverse reactions through toxicity tests in animal models and a few human studies. Hence, *Moringa oleifera* polyphenols may demonstrate tremendous potential in the search for new bioactive compounds to explore for neuroprotective drug discovery and development [83].

Some studies have confirmed that *Moringa oleifera* has a neuroprotective effect [84,85,86,87,88]; as far as we know, nevertheless, no studies have yet described and determined the potential effective neuroprotection components. Quercetin and kaempferol derivatives are the main polyphenols in *Moringa oleifera* leaves. Many studies have reported that these two kinds of compounds have neuroprotective effects [89,90]. What is worth considering is whether these two kinds of compounds play a major role in the neuroprotective effect of polyphenols in *Moringa oleifera* leaves. Therefore, in this part, two representative compounds, isoquercetin and astragaline, which accounted for a relatively high proportion in these two kinds of compounds in *Moringa oleifera* leaf polyphenols, were selected to study the neuroprotective activity in order to explore their role in the neuroprotective activity of *Moringa oleifera* leaves.

To further study the relationship between the MLPE and the two main individual compounds, and to evaluate its protective effect on PC-12 cells damaged by H_2_O_2_, PC-12 cells were incubated with different individual compounds or MLPE for 12 h, then treated with a certain concentration of H_2_O_2_ for 12 h. The established HSCCC separation and preparative method for MLPE permitted isoquercitrin and astragalin to be obtained in large quantities, and thus allowed the cell activity assay to be analyzed and compared.

#### 2.9.1. Effect of Polyphenols in MLPE on the Viability of PC-12 Cells

The effects of MLPE, isoquercitrin and astragalin on the viability of PC-12 cells were determined by the conventional MTT reduction assay. The concentration unit of the MLPE on the PC-12 cells was μg/mL, and the concentration of the two individual compounds was μM.

**Figure 3:**
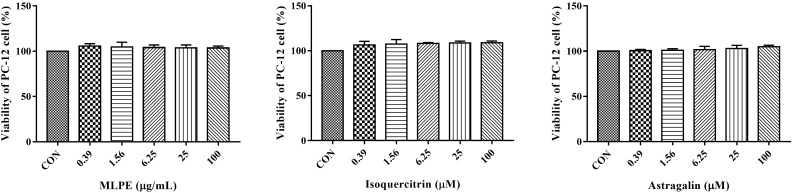
Effect of individual compounds and MLPE on cell viability of PC-12 cells. Cells were pretreated with individual compounds and MLPE for 12 h. Cell viability was assessed by MTT.

The influence of diverse concentrations of individual compounds and MLPE on PC-12 cells is estimated in Figure 3. The results show that compared with the control group, MLPE at the concentrations of 0.39 μg/mL, 1.56 μg/mL, 6.25 μg/mL, 25 μg/mL and 100 μg/mL, individual compounds at the concentrations of 0.39 μM, 1.56 μM, 6.25 μM, 25 μM and 100 μM had little influence on PC-12 cells, and were non-toxic. This is the prerequisite for investigating its effect on damaged cells. All subsequent experiments were performed at these concentrations to evaluate their biological effect.

#### 2.9.2. Effect of H_2_O_2_ on the Viability of PC-12 Cells

**Figure 4:**
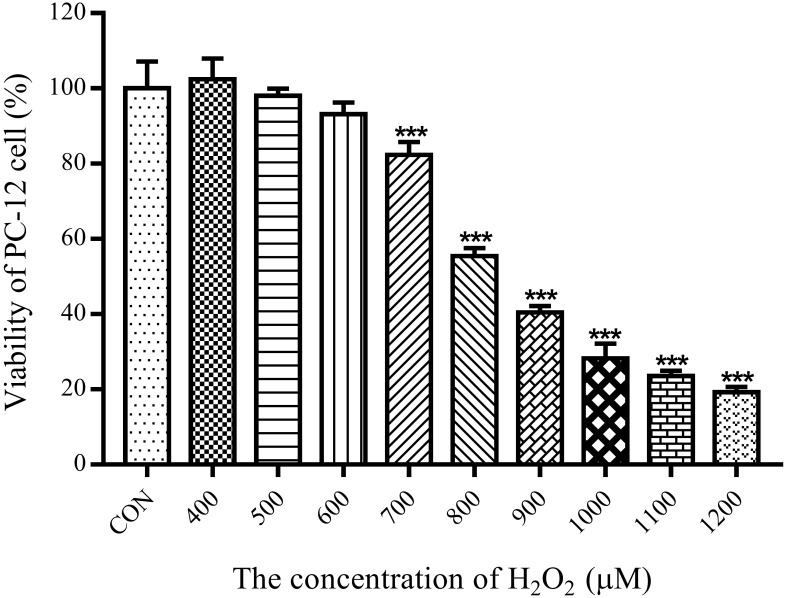
Effect of H_2_O_2_ on cell viability of PC-12 cells. Cells were treated with different concentrations of H_2_O_2_ for 12 h and cell viability was assessed by MTT. *** *p* < 001 vs. control group.

As shown in Figure 4, different concentrations of H_2_O_2_ (400–1200 μM) were added to PC-12 cells and incubated for 12 h to evaluate the relationship between H_2_O_2_ dose and cell viability. The results show that the concentration of H_2_O_2_ visibly decreased cell viability in a dose-dependent manner. Compared with the control group, when the cells were treated with 800 μM H_2_O_2_, the cell survival rate decreased significantly (*p* < 0.001), and the cell viability was 55%, which met the experimental requirements. Therefore, 800 μM was selected for the subsequent experiment.

#### 2.9.3. Protective Effects of Polyphenols in MLPE against Oxidative Stress in PC-12 Cells

**Figure 5:**
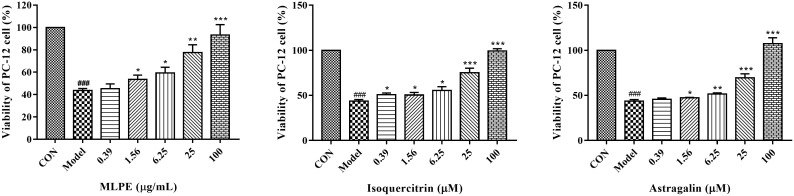
Effect of individual compounds and MLPE on cell viability of H_2_O_2_-induced PC-12 cells. Cells were pretreated with individual compounds and MLPE for 12 h and then treated with H_2_O_2_ (800 µM) for 12 h. Cell viability was assessed by MTT. ### *p* < 0.001 versus control group; * *p* < 0.05, ** *p* < 0.01, and *** *p* < 0.001 vs. H_2_O_2_ group.

Reports on the neuroprotective effect of MLP abound [91,92,93,94,95], in which the object of all of them was MLPE. It remains to be sought for substances with specific functions in MLPE. For instance, the results of Chatchada Sutalangka et al. [96] show that *Moringa oleifera* leaf extract can improve spatial memory and neurodegeneration in CA1, CA2, CA3, and the dentate gyrus of hippocampus, together with the decreased MDA level and AChE activity but increased SOD and CAT activities. However, further explorations concerning the specific active ingredient(s) are still required. Similarly, Woranan Kirisattayakul et al. [97] studied the cerebroprotective effect of the *Moringa oleifera* leaves extract against brain damage and oxidative stress in an animal model of focal ischemic stroke. The results show that *Moringa oleifera* extract can effectively reduce brain dysfunction and injury as well as oxidative stress, but the related active substances were not pointed out in this study. In this study, in order to evaluate the neuroprotective effects of polyphenols in *Moringa oleifera* leaves, MLPE, two representative compounds, isoquercitrin and astragalin, on oxidative stress in PC-12 cells were investigated and compared. As shown in Figure 5, after being treated with MLPE and individual compounds, the viability of PC-12 cells remarkably strengthened in a dose-dependent manner.

Among them, compared with the H_2_O_2_ group, when the concentration of MLPE was 6.25 μg/mL, the cell activity could be restored from 44% to 59%. With the continuous increase in concentration (to 25 μg/mL), the cell activity was significantly restored (78%) (*p* < 0.01). When the cell was treated with 6.25 μM of astragalin, the cell viability was significantly improved compared with the H_2_O_2_ group (*p* < 0.05). However, at the lowest concentration of isoquercitrin (0.39 μM), the cell viability was significantly restored after isoquercitrin treatment (*p* < 0.05), compared with the H_2_O_2_ group. When the concentration of isoquercitrin and astragalin increased to 25 μM, the cell viability was significantly restored to 75% and 69.6% (*p* < 0.001), respectively.

It was verified that MLPE (0.39–100 μg/mL), isoquercitrin and astragalin (0.39–100 μM) could increase the survival rate of PC-12 cells damaged by H_2_O_2_ to varying degrees in a concentration-dependent manner. The EC_50_ value of MLPE was 14.9 μg/mL. Additionally, the EC_50_ values of isoquercitrin and astragalin were 14 μM (6.5 μg/mL) and 33 μM (14.8 μg/mL), respectively. From the semi-maximum effective concentration of these compounds, it is not hard to see that the cell viability order was isoquercitrin > astragalin > MLPE. Among them, the semi-maximum effective concentration of isoquercitrin was far below MLPE. The semi-maximum effective concentration of MLPE was 2.29 and 1.01 times higher than isoquercitrin and astragalin, respectively. The results show that isoquercitrin (quercetin derivative) and astragalin (kaempferol derivative) had strong neuroprotective effects. In the study of Liu et al. [98], isoquercitrin played a neuroprotective role by mitigating the loss of dopamine neurons and reducing the expression of pro-apoptotic signaling molecule. Similarly, the results of Yuan et al. [93] indicate that astragalin also had a strong neuroprotective effect. These studies suggested that polyphenols in *Moringa oleifera* leaves may rely on these two substances to exert neuroprotective effects. Further studies of the exact mechanism are warranted.

In conclusion, the result of the cellular experiment indicate that *Moringa oleifera* leaf polyphenols showed neuroprotective effects, among which isoquercitrin and astragalin may be the potential key active components. Future pharmacological experiments will confirm this hypothesis. This study provided an experimental basis and theoretical support for further studies on the neuroprotective mechanisms of polyphenols in *Moringa oleifera* leaves and may be of great interest from the point of view of developing new health-beneficial products based on polyphenols.

## 3. Materials and Methods

### 3.1. Chemicals and Materials

The organic solvents employed to carry out prep-HSCCC (analytical level) as well as UPLC analysis (chromatographic level) were all purchased from the Chemical Branch of Shandong Yuwang Industrial Co., Yucheng City, China. Standards of kaempferol-3-O-rutinoside, astragalin, and isoquercitrin were purchased from Chengdu Must Bio-Technology Co., Chengdu, China. Standards of 4-caffeoylquinic acid, vitexin and rutin were purchased from Chengdu Chroma Bio-Technology Co., Chengdu, China. The purity of all standards was required to be greater than or equal to 98%.

The PC-12 cell line was provided by the Cell Bank of the Chinese Academy of Sciences (Shanghai, China). Cell culture medium (DMEM), fetal bovine serum (FBS) and penicillin–streptomycin were purchased from Thermo Fisher Scientific Co., Ltd. (St. Wyman, MA, USA). Tetrazolium blue (MTT) and Trypsin were purchased from Beijing Solarbio Science & Technology Co., Ltd. (Solarbio, China).

*Moringa oleifera* leaves for the experiment were identified and provided by SINIFIC (Amadora, Portugal). The fresh leaves (6 years old) were collected from a fertile farm in southeastern Ethiopia with sufficient water sources in July 2019. The picked fresh leaves were dried in the sun, then crushed by a disintegrator and passed through an 80-mesh sieve. The raw material leaf powder was sealed and stored in a polyethylene bag, then refrigerated at 4 °C until used.

### 3.2. Preparation of Polyphenols from Moringa oleifera Leaves by HSCCC

The preparative HSCCC equipment was Model TBE-300 B (Tauto Biotech Co, Shanghai, China) (thereafter, Tauto Biotech). The system has a 20 mL sample loop and three series-connected multi-layer spiral separation columns (with an inner diameter of 1.9 mm and a total volume of 300 mL). The HSCCC system is composed of a UV 2000 D detector (Shanghai Sanotac Scientific Instrument Co., Shanghai, China (thereafter, SSSI)), a TBP-5002 pump and a DC-0506 low-temperature thermostat bath (Tauto Biotech) which was employed in controlling separation temperature. Chromatograms were recorded by employing the EasyChrom-1000 chromatography workstation produced by SSSI.

UPLC used in this work was the Acquity class analysis system, which comprised a Quaternary Solvent Manager (QSM), a Photodiode Array (PDA) Detector (Empower ™ 2 chromatography data software, Milford, MA, USA), and a Sample Manager with Flow through Needle (SM-FTN). The detection wavelength range was 200 nm to 500 nm, and 280 nm was used to detect monomeric polyphenols. The chromatographic column model was Acquity UPLC BEH C18 (50 × 2.1 mm, 1.7 μm), the column temperature was set to 30 °C, and the sample temperature was 10 °C.

### 3.3. Preparation of Moringa oleifera Leaf Phenolic Extract

The preparation of *Moringa oleifera* leaf phenolic extract (MLPE) was performed according to the previous method of our laboratory with slight modification [99,100,101,102]. A total of 500 g of the crushed and sieved *Moringa oleifera* leaves powder was soaked in 1.5 L of 72% ethanol solution, and ultrasonically assisted extraction was performed at 60 °C for 50 min (power 300 W). Then, the extract filtered through a membrane filter (0.45 μm) was collected and dried out at 35 °C under reduced pressure. The aqueous phenolic solution was lyophilized. The powder was thus obtained as crude MLPE, and was stored at −20 °C under darkness prior to further use.

### 3.4. Qualitative Analysis of MLPE Phenolic Compounds by UPLC-Q-Exactive Orbitrap/MS

Qualitative analysis of phenolic compounds in MLPE was carried out by UPLC-Q-Exactive Orbitrap/MS The UPLC conditions were as follows: column, Acquity UPLC BEH C_18_ (50 × 2.1 mm, 1.7 μm); injection volume, 5 μL; flow rate, 0.3 mL/min, detection wavelength, 280 nm; temperature, 30 °C. The gradient elution program adopted two elution solvents, A (water: formic acid; 99.9:0.1, *v*/*v*) and B (acetonitrile): 0 min (A 95%:B 5%), 10 min (A 75%:B 25%) and 18 min (A 0%:B 100%). Mass spectrometry was carried out using the Thermo Scientific Ultimate 3000 system (Thermo Fisher Scientific, Waltham, MA, USA). The optimized conditions of Q-Exactive Orbitrap/MS were as follows: ion transfer tube temperature, 320 °C; heating temperature, 350 °C; gas flow rate, 10 L/min; capillary voltage, −3.0 kV; scanning mode, aux gas pressure, 10 bar; sheath gas pressure, 30 bar; full scan-ddMS^2^ and scanned range, 100–1500 *m*/*z*; HCD collision energy (CE), 20, 30, 40 eV.

### 3.5. Quantitative Analysis of MLPE Phenolic Compounds by UPLC-QqQ/MS

#### 3.5.1. Preparation of Standard Solutions

All standard solutions were freshly prepared. Reference standards of 4-caffeoylquinic acid, vitexin, rutin, isoquercitrin, kaempferol-3-O-rutinoside and astragalin were accurately weighed and dissolved in 72% ethanol to obtain a mixed standard solution with a final concentration of 513 μg/mL, 22.2 μg/mL, 63.6 μg/mL, 120 μg/mL, 43.6 μg/mL and 36.3 μg/mL, respectively. The detection was operated using electrospray negative ionization (ESI^−^). The related conditions of quercetin-acetyl-glycoside and kaempferol-malonyl-glycoside were established according to MLPE. An appropriate amount of hydrochlorothiazide, an internal standard, was accurately weighed, and dissolved with 72% ethanol to prepare the internal standard solution with a concentration of 1 mg/mL.

#### 3.5.2. Preparation of Sample Solutions

A total of 1 g of *Moringa oleifera* leaves was weighed and extracted according to method 2.4. After centrifugation, 900 μL supernatant was added to a 1 mL volumetric flask, the internal standard solution was added, and the solution was shaken well and filtered through a 0.22 μm microporous membrane to obtain sample solution. Due to the limitation of instrument parameters and other reasons, the sample solution was diluted three times.

#### 3.5.3. UPLC-QqQ/MS Instrumentation and Conditions

UPLC-QqQ/MS analysis was carried out on a triple-quadrupole tandem mass spectrometer coupled to an Agilent Acquity UPLC (Agilent, Santa Clara, CA, USA). Chromatographic separations were performed on an Acquity UPLC BEH C18 column (50 mm × 2.1 mm, 1.7 μm). The gradient elution program adopted two elution solvents, A (water: formic acid; 99.9:0.1, *v*/*v*) and B (acetonitrile): 0 min (A 95%:B 5%), 15 min (A 75%:B 25%) and 18 min (A 0%:B 100%). The other major settings were as follows: the flow rate of the mobile phase, 0.3 mL/min; detect wavelength, 280 nm; temperature, 30 °C; the injection volume, 0.5 μL.

The detection of MLPE was operated in the multiple reaction monitoring (MRM) mode using electrospray negative ionization (ESI^−^). The QqQ/MS parameters were set as follows: source temperature 300 °C; nebulizer pressure 45 psi; gas flow 5 L/min; capillary voltage 3.5 KV (ESI^−^); gas temperature 350 °C; cell accelerator voltage 5 V.

#### 3.5.4. Method Validation

According to the signal intensity (peak area) of the MRM transition against different concentration gradients of each standard, the calibration curve was established, and each working solution was injected three times. The limit of detection (LOD) and the limit of quantification (LOQ) were evaluated at the concentrations of signal-to-noise values (S/N) of 3 and 10, respectively.

The precision of the established method was verified via calculating the relative standard deviation (RSDs) of the retention time and peak area by repeating the analysis of the standard samples during the period of intra-day and inter-day. The mixed reference solution was tested six times within the same day with three repeats each time for three consecutive days. Relative standard deviations (RSDs) were used as an index to measure precision. The repeatability of the established method was determined by repeated preparation and analysis of the same sample six times, and the accuracy was further assessed by the recovery experiment. The known quantities of each standard were added to the same sample in sextuplicate, and the same procedure was used for extraction and analysis. To validate stability, the same sample was stored at room temperature, and the injection analysis was repeated at 0, 2, 4, 8, 16 and 24 h. The sample stability was evaluated by the RSDs.

### 3.6. HSCCC Separation

#### 3.6.1. Selection of Solvent System

Combining the structural properties of polyphenols [103,104], minor adjustments were made according to the preliminary laboratory research [47,48,49,105] and the experimental protocols of Kang and Zhu [41,71]. Considering the complexity of the substances in the sample, two representative substances (isoquercitrin and astragalin) were selected from polyphenols in *Moringa oleifera* leaves, and the best solvent system was determined according to their partition coefficient values (*K*). Then, the following six solvent systems were selected based on the partition coefficient values (*K*) of isoquercitrin and astragalin (according to the average polarity of the solvent system): n-hexane-ethyl acetate-methanol-water (HEMWat) (1:20:2:20, *v*/*v*), H_2_O-hexane- ethyl acetate (50:1:50, *v*/*v*), HEMWat (1:5:1:5, *v*/*v*), HEMWat (1:3:1:3, *v*/*v*), HEMWat (3:5:3:5, *v*/*v*), and HEMWat (1:1:1:1, *v*/*v*). The peak area of the sample in the two phases of the solvent system was determined by Acquity H-class UPLC system (Waters, Milford, MA, USA), then the partition coefficient *K* = A_1_/A_2_ (stationary phase/mobile phase) and separation factor (α) = *K*_2_/*K*_1_ (*K*_2_/*K*_1_) were calculated.

The *K* and α values were used as assessment indicators for selecting the two-phase solvent system used to carry out the HSCCC separation. The *K* and α values of target components were estimated through UPLC analysis as follows: In a test tube with a capacity of 10 mL, 2.5 mg of the crude MLPE was dissolved in an equilibrated mixture of 5 mL of the upper phase and 5 mL of the lower phase. The test tube was then plugged and shaken strongly for about one minute to allow the sample between the two phases to equilibrate fully. Next, equal volumes (5 mL) of the two phases were dried out individually, and methanol was used to dilute the residues and UPLC was employed to conduct the analysis.

#### 3.6.2. Preparation of Solvent System and Sample Solution

In this research, by shaking it vigorously under room temperature many times, the chosen two-phase solvent system was fully equilibrated in the separation funnel. Before being used, the two phases were broken apart and degassed through sonication for about 15 min. In addition, 100 mg of the MLPE was dissolved in 10 mL of upper phase to prepare the sample solution for the HSCCC separation.

#### 3.6.3. HSCCC Separation Procedure

During each HSCCC separation process, firstly, the multi-layer spiral chromatographic column was completely filled with the upper phase (stationary phase) and then the rotational speed of the device was adjusted to 950 rpm. At the same time, this study pumped the lower phase (mobile phase) into the column’s head at the flow rate of 2.5 mL per minute. When a clear mobile phase flows out from the tail end, it indicates that the two-phase solvent has reached hydrodynamic equilibrium in the instrument, and the sample can be injected through the injection valve at this time. Then, a UV detector was used to constantly monitor the effluent at the tail end at 280 nm, the chromatogram was recorded, and the retention of the stationary phase computed. After that, the generated fractions were dried out at 35 °C under reduced pressure and freeze dried, and the powder was kept in a dark environment at −20 °C.

#### 3.6.4. UPLC–MS Analysis of HSCCC Peak Fractions and MS Identification of Individual Polyphenols

UPLC with quadrupole Exactive Orbitrap/MS was employed to qualitatively analyze the individual polyphenols. The injection volume was 1 μL. Two elution solvents, A (water:formic acid; 99.9:0.1, *v*/*v*) and B (acetonitrile) were used with the gradient elution program. Both the optimized conditions of UPLC and MS are the same as 2.5.

### 3.7. Protective Effects of Individual Compounds and MLPE against Oxidative Stress in PC-12 Cells

#### 3.7.1. Cell Culture Conditions

PC-12 cells were cultured in DMEM containing 10% FBS, 100 μg/mL penicillin and 100 μg/mL streptomycin in a 5% CO_2_ humidified atmosphere at 37 °C. The growth medium was replaced once a day. The PC-12 cells were digested and passaged by routine enzyme.

#### 3.7.2. Effects of Individual Compounds and MLPE on the Viability of PC-12 Cells

To evaluate the effect of isolated polyphenols and MLPE on cell viability, 1 × 10^4^ cells/mL PC-12 cells were seeded in flat-bottom 96-well plates (100 µL/well), and incubated in a Thermo 311 CO_2_ incubator (Thermo Fisher Scientific, TFS) for 12 h. Cells were then treated with different concentrations of individual compounds and MLPE for 12 h. Cell viability was measured by MTT assay: MTT was dissolved in 1 × sterile PBS at 5 mg/mL concentration. Then, 10 µL of MTT reagent was added to each well of the cell culture plate, shaken continuously for 1 min, and then incubated at 37 °C for 4 h. Whereafter, the culture medium was carefully removed, and 100 µL of dimethyl sulfoxide was added to each well to dissolve the formazan crystal, and OD_570_, OD_630_ was measured with an enzyme-labeling instrument (Bio-Rad iMARK, Berkeley, CA, USA). The experiment was repeated three times. The cell viability was calculated as the percentage of reduced MTT, as follows:

Cell viability (%) = (A_570_ − A_630_) of individual compounds and MLPE treatment/(A_570_ − A_630_) of the control × 100%

#### 3.7.3. Effect of Hydrogen Peroxide (H_2_O_2_) on the Viability of PC-12 Cells

The dose–response (400 to 1200 µM) of H_2_O_2_′s cytotoxic effect was measured with MTT assay. The same experimental procedure was applied as in Section 2.8.2.

#### 3.7.4. Protective Effects of Individual Compounds and MLPE against Oxidative Stress in PC-12 Cells

On the basis of preliminary experiments, the final process conditions were confirmed. The procedure was also the same as in Section 2.8.2, with the exception that compound concentrations of 0.39, 1.56, 6.25, 25 and 100 μM (the concentration unit of MLPE is μg/mL) were adopted and incubated in a 5% CO_2_ humidified atmosphere at 37 °C for pre-treatment for 12 h. Then, the medium was then carefully removed, and 100 μL of H_2_O_2_ (800 μM, final concentration) with different concentrations of individual compounds and MLPE (0.39, 1.56, 6.25, 25 and 100 μM or μg/mL) were added to incubate for 12 h. The viability of cells was estimated by MTT assay.

### 3.8. Statistical Analysis

GraphPad Prism 7.0 software (GraphPad, San Diego, CA, USA), Origin 9.0 software (OriginLab, Northampton, MA, USA) and SPSS 21.0 software (IBM, Armonk, NY, USA) were used for analysis of variance and to evaluate the significance of differences. *p* < 0.05 was considered statistically significant.

## 4. Conclusions

In this study, the phenolic composition of *Moringa oleifera* leaves was qualitatively and quantitatively determined by UPLC-Q-Exactive Orbitrap/MS and UPLC-QqQ/MS, respectively. HSCCC was used to separate the main polyphenols in *Moringa oleifera* leaves for the first time. Additionally, the efficient separation of the main polyphenols was achieved by merely one-step HSCCC separation in the shortest time (120 min) with the optimal suitable solvent system. The isolated flavonols, such as quercetin-acetyl-glycoside and kaempferol-malonyl-glycoside, are not commercially available. Meanwhile, the analysis of cellular antioxidant activity showed that the isolated polyphenols and MLPE had a strong neuroprotective effect, suggesting that *Moringa oleifera* leaf may act as a natural source of neuroprotective agent.

## Figures and Tables

**Table 1:** MRM parameters, cone voltage (CV) and collision energy (CE) for each standard measured.

Standards/Equivalent Compounds	Precursor Ion (*m*/*z*)	Product Ion (*m*/*z*)	Fragmentor (V)	Collision Energy (eV)	Polarity
4-Caffeoylquinic acid	353.2	173.2	120	15	Negative
Vitexin	431.2	311.1	200	20	Negative
Rutin	609.2	300.1	270	36	Negative
Isoquercitrin	463.1	300.1	200	24	Negative
Kaempferol-3-O-rutinoside	593.2	285.2	230	35	Negative
Astragalin	447.1	284.1	210	28	Negative
Quercetin-acetyl-glycoside	505.2	300.1	200	25	Negative
Kaempferol-malonyl-glycoside	533.1	489.2	150	5	Negative

**Table 2:** List of tentative compounds identified in *Moringa oleifera* leaves by UPLC-Q-Exactive-MS/MS under negative ionization.

No.	Compounds	t_R_/min	[M–H]^−^	MS/MS	Theoretical Mass	Error (ppm)	Formula
1	Caffeic acid	0.53	178.9769	89.0228/161.0444	180.0422	−0.82	C_9_H_8_O_4_
2	Quinic acid	0.66	190.9276	85.0278/127.0387	192.0633	−0.90	C_7_H_12_O_6_
3	Malic acid	0.73	133.0129	115.0022/71.0122	134.0215	−0.31	C_4_H_6_O_5_
4	Glucomoringin	0.92	570.0964	96.9585/259.0131	571.1029	−2.58	C_20_H_29_NO_14_S_2_
5	Vanillin glucoside	1.48	315.0724	153.0545/108.0201	316.1158	−0.82	C_14_H_20_O_8_
6	4-Caffeoylquinic acid	1.63	353.0880	191.0552/179.0340	354.0951	−0.45	C_16_H_18_O_9_
7	Vanillin glucoside	2.01	315.0724	153.0545/123.0438	316.1158	−0.72	C_14_H_20_O_8_
8	3-Caffeoylquinic acid	2.34	353.0880	191.0552/179.0340	354.0951	0.51	C_16_H_18_O_9_
9	4-Coumaroylquinic acid	3.16	337.0931	163.0389/119.0488	338.1002	−1.57	C_16_H_18_O_8_
10	3-Coumaroylquinic acid	3.68	337.0931	163.0389/119.0488	338.1002	2.94	C_16_H_18_O_8_
11	Feruloylquinic acid	4.74	367.1036	193.0498/134.0359	368.1107	1.10	C_17_H_20_O_9_
12	Vicenin-2	6.74	593.1516	353.0668/383.0775	594.1585	−0.53	C_27_H_30_O_15_
13	Vitexin	8.52	431.0984	311.0563/341.0666	432.1056	0.14	C_21_H_20_O_10_
14	Rutin	8.62	609.1464	300.0276/343.0455	610.1534	1.02	C_27_H_30_O_16_
15	Isoquercitrin	8.76	463.0887	300.0276/343.0464	464.0955	−0.99	C_21_H_20_O_12_
16	Quercetin-acetyl-glycoside	9.41	505.0993	300.0276/343.0455	506.1060	−0.32	C_23_H_22_O_13_
17	Quercetin-malonyl-glucoside	9.41	549.0888	300.0276	550.0959	−1.06	C_24_H_22_O_15_
18	Kaempferol-3-O-rutinoside	9.52	593.1516	285.0405/327.0510	594.1585	−0.53	C_27_H_30_O_15_
19	Isolariciresinol glucoside	9.53	521.2034	341.1398/101.0228	522.2101	0.91	C_26_H_34_O_11_
20	Astragalin	9.88	447.0936	284.0327/327.0513	448.1006	−0.20	C_21_H_20_O_11_
21	Kaempferol-acetyl-glycoside	10.7	489.1042	284.0328/327.0495	490.1111	−0.43	C_23_H_22_O_12_
22	Kaempferol-malonyl-glycoside	10.7	533.1724	285.0404/255.0295	534.1010	1.16	C_24_H_22_O_14_

**Table 3:** Calibration curve, LOD and LOQ of standards.

Compounds	Regression Equation	r	Linear Range (μg/mL)	LOD (μg/mL)	LOQ (μg/mL)
4-Caffeoylquinic acid	y = 2.384x + 0.1378	0.9993	6.413–410.4	0.05388	0.1796
Vitexin	y = 10.70x + 0.0285	0.9996	0.2775–17.76	0.01281	0.04271
Rutin	y = 6.293x + 0.0261	0.9993	0.7950–50.88	0.02228	0.07425
Isoquercitrin	y = 9.22x + 0.1726	0.9995	1.500–96.0	0.01488	0.04959
Kaempferol-3-O-rutinoside	y = 2.630x + 0.0231	0.9997	0.5450–34.88	0.05225	0.1742
Astragalin	y = 11.86x + 0.1738	0.9991	0.4538–29.04	0.01046	0.03486

**Table 4:** Precision, repeatability and stability of standards.

Compounds	Precision (%)		Repeatability	Stability
	Intra-Day RSD	Inter-Day RSD	RSD (%)	RSD (%)
4-Caffeoylquinic acid	2.9	3.4	2.5	3.4
Vicenin-2	1.5	1.1	0.6	0.9
Vitexin	3.6	3.8	2.3	2.3
Rutin	0.3	0.3	0.4	4.8
Isoquercitrin	0.03	0.1	0.5	3.4
Kaempferol-3-O-rutinoside	0.6	0.8	4.8	4.6
Astragalin	1.1	1.1	1.2	2.9

**Table 5:** Recoveries of compounds in MLPE (*n* = 6).

Compounds	Initial Amount (mg)	Added Amount (mg)	Detected Amount (mg)	Recovery (%)	RSD (%)
4-Caffeoylquinic acid	1.045	1.054	2.032	96.8	2.1
	1.048	1.054	2.060	98.0	
	1.043	1.054	2.021	96.4	
	1.046	1.054	2.016	96.0	
	1.046	1.054	2.132	101.5	
	1.043	1.054	2.049	97.7	
Vitexin	0.1693	0.1884	0.3410	95.3	4.1
	0.1708	0.1884	0.3769	104.9	
	0.1650	0.1884	0.3382	95.7	
	0.1710	0.1884	0.3537	98.4	
	0.1691	0.1884	0.3679	102.9	
	0.1673	0.1884	0.3425	96.3	
Rutin	1.244	1.271	2.484	98.8	1.9
	1.258	1.271	2.519	99.6	
	1.195	1.271	2.396	97.2	
	1.212	1.271	2.405	96.9	
	1.218	1.271	2.541	102.1	
	1.245	1.271	2.493	99.1	
Isoquercitrin	1.145	1.190	2.387	102.2	2.5
	1.178	1.190	2.337	98.7	
	1.126	1.190	2.218	95.8	
	1.140	1.190	2.242	96.2	
	1.145	1.190	2.276	97.5	
	1.158	1.190	2.266	96.5	
Kaempferol-3-O-rutinoside	0.6031	0.6099	1.236	101.9	3.2
	0.6018	0.6099	1.183	97.6	
	0.5125	0.6099	1.159	103.3	
	0.5366	0.6099	1.128	98.4	
	0.5578	0.6099	1.112	95.2	
	0.5744	0.6099	1.141	96.3	
Astragalin	0.1741	0.1848	0.3733	104.0	3.9
	0.1804	0.1848	0.3552	97.3	
	0.1736	0.1848	0.3712	103.6	
	0.1769	0.1848	0.3443	95.2	
	0.1742	0.1848	0.3519	98.0	
	0.1788	0.1848	0.3477	95.6	

**Table 6:** Contents of 10 polyphenolic compounds in MLPE.

Compounds	t_R_(min)	Contents (mg/g DW)
4-Caffeoylquinic acid	3.7	1.985 ± 0.008
Vitexin	7.8	0.3415 ± 0.005
Rutin	7.9	2.267 ± 0.006
Isoquercitrin	8.2	2.293 ± 0.005
Quercetin-acetyl-glycoside	9.1	2.456 ± 0.004
Kaempferol-3-O-rutinoside	9.3	1.206 ± 0.003
Astragalin	9.6	0.4007 ± 0.002
Kaempferol-malonyl-glycoside	10.8	0.1254 ± 0.003

**Table 7:** Partition coefficient values (*K*) and separation factors (α) of six solvent systems.

Solvent Systems (*V*/*V*)	Isoquercitrin (*K*_1_)	Astragalin (*K*_2_)	α
HEMWat (1:20:2:20, *v*/*v*)	1.00	1.17	1.17
H_2_O-hexane- ethyl acetate (50:1:50, *v*/*v*)	2.20	106.9	-
HEMWat (1:5:1:5, *v*/*v*)	1.01	0.79	1.28
HEMWat (1:3:1:3, *v*/*v*)	1.01	0.77	1.31
HEMWat (3:5:3:5, *v*/*v*)	1.01	54.49	-
HEMWat (1:1:1:1, *v*/*v*)	1.00	28.55	-

**Table 8:** Retention time (TR) of individual polyphenols and their major ions observed in the MS/MS spectra.

No.	T_R_ (min)	[M–H]^−^	MS/MS	Formula	Compounds
1	3.7	353.0880	191.0552/179.0340	C_16_H_18_O_9_	4-Caffeoylquinic acid
2	7.9	609.1464	300.0276	C_27_H_30_O_16_	Rutin
3	9.1	505.0993	300.0276	C_23_H_22_O_13_	Quercetin-acetyl-glycoside
4	9.2	593.1516	285.0405	C_27_H_30_O_15_	Kaempferol-3-O-rutinoside
5	7.7	431.0984	311.0563/341.0666	C_21_H_20_O_10_	Vitexin
6	8.2	463.0887	300.0276	C_21_H_20_O_12_	Isoquercitrin
7	9.5	447.0936	284.0327/255.0300	C_21_H_20_O_11_	Astragalin
8	10.8	533.1724	285.0404	C_24_H_22_O_14_	Kaempferol-malonyl-glycoside

## Data Availability

Not applicable.

## Sample Availability

Samples of the compounds are available from the authors.
